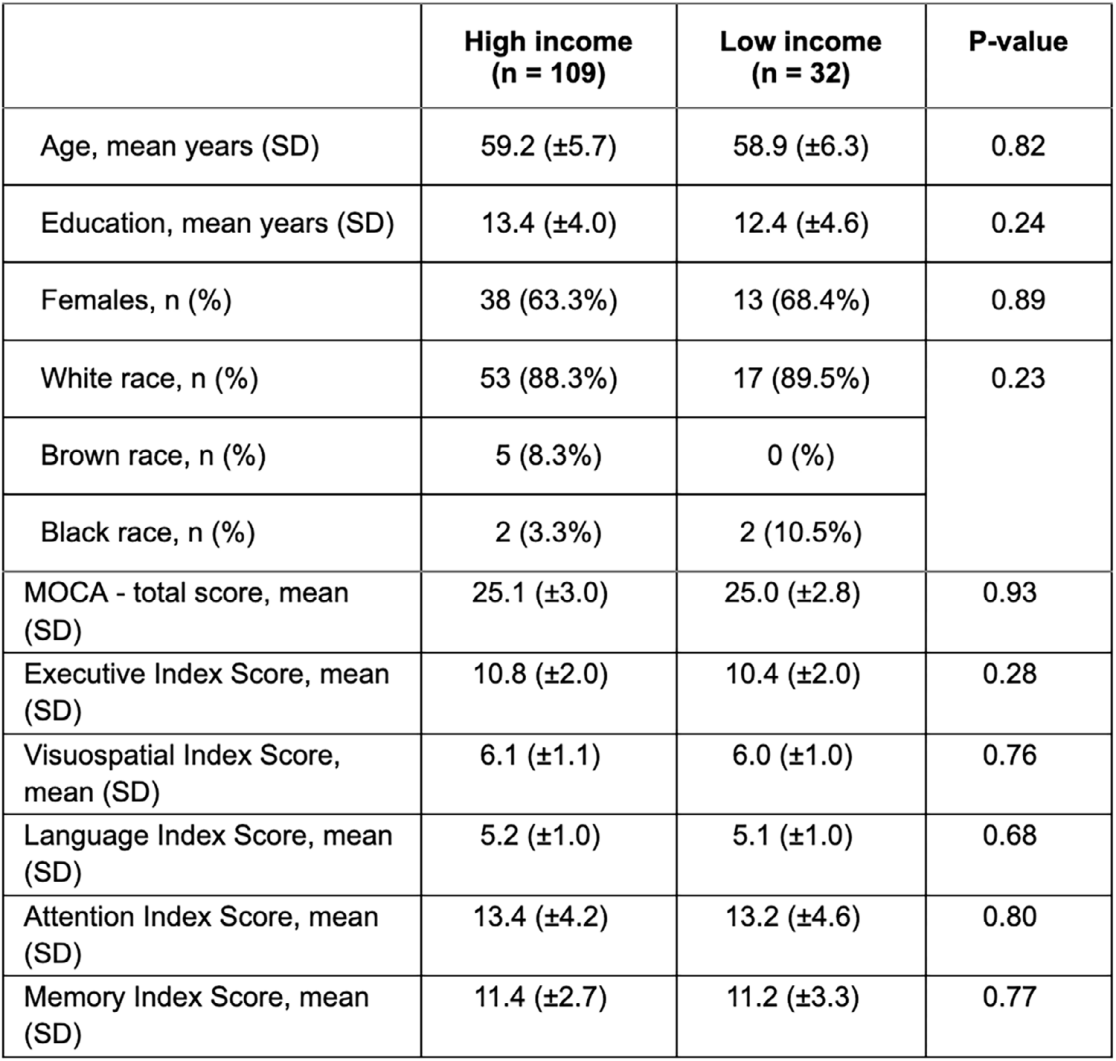# Executive functions and language domains are vulnerable to Social Determinants of Health: A cognitive analysis from the PROMOTE trial

**DOI:** 10.1002/alz70860_105692

**Published:** 2025-12-23

**Authors:** Jordana Simoes Braga, Thaís Secchi, Danielle Pereira, Francine Würzius Quadros, Aline Palmeira Pires, Magda Martins, Bruna Jaeger, Franciele Pereira Santos, Wyllians Vendramini Borelli, Sheila O Martins

**Affiliations:** ^1^ UFRGS, Gravataí, Brazil; ^2^ Memory center, Hospital Moinhos de Vento, Brazil, Porto Alegre, Brazil; ^3^ Universidade Federal do Rio Grande do Sul, Porto Alegre, Rio Grande do Sul, Brazil; ^4^ Federal University of Rio Grande do Sul (UFRGS), Porto Alegre, Brazil

## Abstract

**Background:**

Social determinants of health (SDH) frame a complex interplay between environmental factors that increase the risk of many health conditions, including dementia. However, it is unclear whether SDH impairs cognitive domains differently. This study aimed to investigate the impact of SDH across distinct cognitive domains in a South American population, expanding current understanding predominantly based on European and North American cohorts.

**Method:**

Baseline data from the PROMOTE trial, conducted in a Brazilian cohort, was collected between 2023 and 2022. Participants were clinically evaluated and underwent the Montreal Cognitive Assessment (MoCA), with scores divided into subscores for six cognitive domains: Memory Index Score (MIS), Executive Index Score (EIS), Attention Index Score (AIS), Language Index Score (LIS), Visuospatial Index Score (VIS), and Orientation Index Score (OIS). SDH variables included years of education, ethnicity, family income, and neighborhood income. Regression models were used to evaluate the impact of SDH on total MoCA scores and subscores, adjusted for age and sex.

**Result:**

Data from 147 participants (mean age: 59 years; mean years of education: 13.2) were analyzed. The majority of participants were White (*n* = 139), while 12 were non‐White (Tab. 1). Total MOCA scores were not associated with any SDH. However, regression analysis revealed that SDH had distinct associations with MoCA subscores. Years of education were significantly associated with EIS (β = 0.11, *p*‐adjusted = 0.002). Additionally, lower family income was significantly associated with EIS (β = ‐0.32, *p* = 0.04). For LIS, neighborhood income was significantly associated (β = 0.03, *p* = 0.04). In contrast, no significant associations were found between racial origin and MoCA subscores.

**Conclusion:**

SDH independently influences specific cognitive domains. Education and family income significantly impact the Executive Index, while neighborhood income affects the Language domain. These findings underscore the critical role of socioeconomic factors in cognitive health and the nuanced ways in which SDH affect distinct cognitive domains. Racial origin did not show a significant influence, emphasizing the importance of targeting socioeconomic interventions to address disparities in cognitive outcomes.